# Merkel Cell Polyomavirus DNA in Persons without Merkel Cell Carcinoma

**DOI:** 10.3201/eid1509.081575

**Published:** 2009-09

**Authors:** Ulrike Wieland, Cornelia Mauch, Alexander Kreuter, Thomas Krieg, Herbert Pfister

**Affiliations:** Institute of Virology, Koeln, Germany (U. Wieland, H. Pfister); Department of Dermatology, Koeln (C. Mauch, T. Krieg); Department of Dermatology, Bochum, Germany (A. Kreuter)

**Keywords:** Merkel cell polyomavirus, MCPyV, Merkel cell carcinoma, human papillomavirus, HPV, human immunodeficiency virus, HIV, viruses, dispatch

## Abstract

Merkel cell polyomavirus (MCPyV) DNA was detected in 88% of Merkel cell carcinomas in contrast to 16% of other skin tumors. MCPyV was also found in anogenital and oral samples (31%) and eyebrow hairs (50%) of HIV-positive men and in forehead swabs (62%) of healthy controls. MCPyV thus appears to be widespread.

Merkel cell polyomavirus (MCPyV) was recently discovered in Merkel cell carcinomas (MCC), rare but aggressive skin cancers (*1*). MCPyV DNA has been detected in the majority of MCC and less commonly in other skin tumors and healthy skin (*1*–*6*). To help determine if MCPyV might be widespread in the general population, we conducted a retrospective study and tested MCC as well as healthy and lesional skin and mucosa samples of immunocompetent and immunosuppressed persons without MCC for MCPyV-DNA.

## The Study

All samples (n = 355) were analyzed by hot-start single-round LT3-PCR (sPCR) and nested LT1/M1M2-PCR (nPCR) by using primers described previously (*1*) (experimental details on DNA isolation, controls, and PCR conditions are available from U.W.). Because analytical sensitivities of sPCR and nPCR were 1,000 copies of cloned LT3-DNA and 10 copies of cloned LT1-DNA per assay, samples positive by both PCRs probably had higher viral loads than those positive only by nPCR. The sPCR- or nPCR-products of 19 MCC and 48 non-MCC samples were sequenced and were MCPyV specific.

MCPyV DNA was detectable in 30/34 (88%) MCC biopsies and in 5/5 (100%) MCC metastases by nPCR, and in 68% and 80%, respectively, by sPCR. MCPyV DNA was found by nPCR only in 1/13 (7.7%) whole blood samples of MCC-patients. The patient with MCPyV-positive blood had positive sPCR/nPCR results for MCC and positive nPCR results for a second sample taken from the previous MCC site. Of 5 further non-MCC biopsy samples from MCC patients, 1 skin sample from a patient with unspecific dermatitis was positive by nPCR.

MCPyV DNA was traceable only by nPCR in 10/61 (16%) biopsy samples of different non-MCC skin tumors and in 8/34 (24%) of perilesional, clinically, and histologically healthy skin samples from 56 immunocompetent patients (*7*) without MCC (Table 1). MCPyV DNA status was identical in 30/32 pairs of tumor and corresponding perilesional skin samples (negative/negative in 24, positive/positive in 6, divergent in 2 pairs). MCPyV was found significantly more often in MCC (n = 34) than in non-MCC skin tumors (n = 61) or perilesional skin biopsies (n = 34) (p<0.001; χ^2^ test).

**Table 1:** MCPyV DNA in biopsies of immunocompetent patients without MCC*

Histologic diagnosis	No. samples	No. (%) MCPyV-positive by nested PCR
Papilloma/wart	4	0 (0)
Actinic keratosis	7	0 (0)
Keratoacanthoma	7	3 (43)
Squamous cell carcinoma	6	1 (17)
Bowen disease/carcinoma	4	1 (25)
Basal cell carcinoma	21	3 (14)
Malignant melanoma	12	2 (17)
All skin tumors	61	10 (16)
Perilesional healthy skin	34	8 (24)

*MCPyV, Merkel cell polyomavirus; MCC, Merkel cell carcinoma. All samples shown were negative for Merkel cell polyomavirus in single-round PCR. Six MCPyV-positive perilesional samples had an MCPyV-positive lesional counterpart (3 basal cell carcinomas, 2 keratoacanthomas, 1 squamous cell carcinoma); 1 MCPyV-positive perilesional biopsy had a MCPyV-negative counterpart (basal cell carcinoma); and of 1 MCPyV-positive perilesional biopsy the lesional counterpart was not available. For origin of samples see (*7*).

Mucosal samples were available from 79 HIV-infected men who have sex with men (HIV-MSM) (without MCC) participating in an anogenital dysplasia/human papillomavirus (HPV) screening program (*8*). MCPyV DNA was detectable in 37/120 (31%) of all mucosal (anal, penile, oral) samples by nPCR and in 10/120 (8%) by sPCR (Table 2). In anal samples, MCPyV DNA positivity was lowest in anal cancer tissues (14% by nPCR), followed by dysplasias (26%), swabs with normal cytology (30%), and benign lesions (33%). Similar values were found for penile samples; 29% of dysplasias, 33% of benign lesions, and 50% of normal swabs were MCPyV DNA positive. In oral samples, MCPyV DNA was detected in 39% of normal swabs, in 0% of benign lesions, and in 50% of carcinomas in situ. MCPyV DNA positivity was not associated with the presence of mucosal premalignant and malignant lesions (p = 0.597; n = 120; 1-sided analysis of variance test), in contrast to positivity for high-risk (HR)-alpha-HPV, the established etiologic agents of these lesions (p = 0.001; n = 120) (Table 2). MCPyV DNA positivity was not significantly different in mucosal samples from HIV-MSM with CD4 counts below or above 200/μL (29% vs. 32%; p = 0.839; n = 120; χ^2^ test). For HR-HPV, a trend for a higher detection rate in patients with CD4 counts <200/μL could be observed (80% vs. 67%; p = 0.145; n = 120) (Table 2). In 7 cerebrospinal fluid samples from HIV-MSM with central nervous system problems, MCPyV DNA was not detected.

**Table 2:** MCPyV DNA and human papillomavirus DNA in samples of HIV-positive men without Merkel cell carcinoma*

Diagnosis	No. samples	No. (%) MCPyV-positive by nPCR	No. (%) MCPyV-positive by sPCR	No. (%) HPV-positive	No. (%) HR-HPV–positive†
Normal‡ anogenital/oral swabs	49	18 (37)	7 (14)	32 (65)	31 (63)
CD4 <200/μL	18	6 (33)	3 (17)	13 (72)	13 (72)
CD4 >200/μL	31	12 (39)	4 (13)	19 (61)	18 (58)
Anal	20	6 (30)	3 (15)	20 (100)	20 (100)
Penile	6	3 (50)	3 (50)	4 (67)	3 (50)
Oral	23	9 (39)	1 (4)	8 (35)	8 (35)
Benign papilloma/acanthoma	21	6 (29)	2 (10)	20 (95)	10 (48)
Anal	12	4 (33)	2 (17)	12 (100)	5 (42)
Penile	6	2 (33)	0 (0)	6 (100)	2 (33)
Oral	3	0 (0)	0 (0)	2 (67)	3 (100)
Dysplasia/carcinoma in situ	43	12 (28)	1 (2)	41 (95)	38 (88)
Anal intraepithelial neoplasia	27	7 (26)	1 (4)	26 (96)	26 (96)
Penile intraepithelial neoplasia	14	4 (29)	0 (0)	13 (93)	12 (86)
Oral carcinoma in situ	2	1 (50)	0 (0)	2 (100)	0 (0)
Anal cancer	7	1 (14)	0 (0)	7 (100)	7 (100)
All anal, penile, or oral samples	120	37 (31)	10 (8)	100 (83)	86 (72)
CD4 <200/μL	45	13 (29)	5 (11)	38 (84)	36 (80)
CD4 >200/μL	75	24 (32)	5 (7)	62 (83)	50 (67)
Plucked eyebrow hairs	14	7 (50)	5 (36)	14 (100)	0 (0)
Cerebrospinal fluid	7	0 (0)	0 (0)	ND	ND

*MCPyV, Merkel cell polyomavirus; nPCR, nested PCR; sPCR, single-round PCR; HPV, human papillomavirus; HR, high-risk alpha-HPV. All samples were tested for beta-HPV. Eyebrow hairs were additionally tested for alpha-HPV, and all were positive. Alpha- and beta-HPV DNA were determined by PCRs as previously described (*8*). †HR types comprise HPV16, 18, 26, 31, 33, 35, 39, 45, 51, 52, 53, 56, 58, 59, 66, 68, 73, 82. For details of the patient-collective see (*8*). ‡Normal swabs were those negative for intraepithelial lesion or malignancy.

In an immunodeficient patient with WILD syndrome (warts, immunodeficiency, lymphedema, anogenital dysplasia) (*9*), MCPyV DNA was found by nPCR and sPCR on the abdominal, thigh, perianal, and vulvar skin, and by nPCR in the vagina, cervix, and intraanal canal (20/27 swabs were nPCR- and 4/27 sPCR-positive). A whole blood sample and 5 papilloma biopsies were MCPyV DNA negative. MCPyV DNA was detected by nPCR in the cellular pellet but not in the supernatant of a urine sample of the patient with WILD syndrome. The presence of MCPyV DNA in the cellular pellet was probably caused by MCPyV- positive urogenital cells flushed into the urine.

MCPyV DNA was not found in 13 BKPyV DNA positive urine samples from 11 renal-transplant recipients without MCC. MCPyV DNA was detected by nPCR in 7/14 (50%) of plucked eyebrow hairs of 14 HIV-MSM and by sPCR in 5/14 (36%) (Table 2) as well as in eyebrow hairs of the patient with WILD syndrome (nPCR-positive). Skin swabs covering 20 cm^2^ of the forehead were taken from 13 healthy immunocompetent male adults (*10*), and MCPyV DNA was detected by nPCR in 8/13 (62%) and by sPCR in 5/13 (38%).

## Conclusions

Using nested PCR, we found MCPyV DNA in 88% of in samples from persons with MCC. In 68%, viral DNA load was high enough to be detectable by sPCR. This finding is similar to detection rates reported before (54%–89%) (*1*–*6*) and confirms the association of MCPyV with MCC.

The MCPyV positivity of 16% in non-MCC skin tumors was significantly lower than in MCC; MCPyV DNA was only detectable by nPCR, pointing to lower viral loads than in MCC. Similar to our results, MCPyV DNA has been found in 12.5% of basal cell carcinomas and viral load was 4-log lower than in MCC (*2*). In other studies, MCPyV DNA was detected in 13% of squamous cell carcinomas and only in 1 keratoacanthoma of 156 non-melanoma skin cancers (*4*,*6*). The relatively low detection rate of MCPyV in non-MCC skin tumors, similar to that in healthy, perilesional skin, suggests that MCPyV probably does not play a role in the development of non-MCC skin tumors.

HR-alpha-HPV induces anogenital dysplasia/cancer and HIV-MSM have a strongly increased risk for developing these lesions (*11*). In anogenital samples of HIV-MSM, MCPyV DNA was less common in premalignant and malignant lesion samples than in benign samples or samples with normal cytology. Thus, it is unlikely that MCPyV plays a role in the development of anogenital dysplasia/cancer in HIV-MSM. In contrast to HR-alpha-HPV, MCPyV recovery was not increased in HIV-MSM with advanced immunodeficiency.

MCPyV DNA was detected only once in hematolymphoid tissue and never in donated blood (*5*,*12*). Similarly, we could not detect MCPyV DNA in 12/13 blood samples obtained from patients with MCC and in the blood sample of the patient with WILD syndrome, who was MCPyV positive in numerous other samples. MCPyV DNA was not found in BKPyV-positive urine samples from renal transplant recipients or in the cell-free urine supernatant of the patient with WILD syndrome.

In normal skin, MCPyV DNA has been identified before by PCR and Southern blot in 1/6 biopsies (*1*) but not in 15 samples when real-time PCR was used (*4*). Surprisingly, we found MCPyV DNA by sPCR in 38% and by nPCR in 62% of area-wide skin swabs from the forehead of healthy controls. Furthermore, MCPyV DNA was found in 14% and 37% of normal mucosa swabs of HIV-MSM by sPCR and nPCR, respectively. Since Merkel cells are found within the basal layer of the epidermis (*13*), it is unlikely that they are collected in surface-swabs. This observation suggests that the detected MCPyV DNA either represents cell-free virus that may have been produced in Merkel cells or virus in superficial keratinocytes. Thirty-six percent (sPCR) and 50% (nPCR) of eyebrow hairs of HIV-MSM carried MCPyV DNA. High concentrations of Merkel cells were described in the bulge region of hair follicles (*14*). Hair bulbs have been suggested as a reservoir for beta-HPVs (*15*), and this may also be true for MCPyV. Our data demonstrate a widespread distribution of MCPyV in normal skin, mucosa and hair bulbs, although MCPyV does not reach the magnitude found for ubiquitous beta-HPV (*10*,*15*). Our nonpopulation based data need to be confirmed in cross-sectional studies, but it is likely that MCPyV is prevalent in the general population.
